# Reported Beliefs About COVID-19 Vaccines Among Unvaccinated Hispanic Adults Utilizing a Free Clinic in Orlando, Florida

**DOI:** 10.7759/cureus.35255

**Published:** 2023-02-21

**Authors:** Thomas M Knapp, Caridad Hernandez, Jeffrey Simpson, Kailee Hernandez, Catalina Esguerra, Bieu T Mach, Lindsay A Taliaferro

**Affiliations:** 1 Medicine, University of Central Florida, Orlando, USA

**Keywords:** qualitative studies, immigrant health, qualitative semi-structured interviews, hispanic health, covid-19 vaccine hesitancy

## Abstract

Background: Subgroups of the general population including Hispanic/Latinx individuals report higher rates of COVID-19 vaccine hesitancy than non-Hispanic White individuals. The purpose of this study was to identify factors that influence attitudes toward COVID-19 vaccines among unvaccinated Hispanic adults utilizing a free community clinic in Orlando, Florida, USA.

Methods: From May 2021 to July 2021, we used convenience sampling to recruit 20 self-identified Hispanic adults who were unvaccinated to complete an individual, semi-structured interview. Interview questions were derived from constructs from the Health Belief Model. Interviews were audio-recorded, transcribed, translated (when necessary), and qualitatively analyzed using inductive content analysis to identify recurring themes.

Results: Of the 20 participants in this study, 65% were female (n=13) and they ranged from 21 to 73 years of age (median age =42.5). We identified three primary themes in participant responses regarding their beliefs about COVID-19 vaccines. Primary theme 1: trust and clarity of COVID-19 vaccine information, with subthemes (1a) source trustworthiness, and (1b) clarity of COVID-19 vaccine information. Primary theme 2: personal contextual factors, with subthemes (2a) underlying health conditions, (2b) personal experiences with COVID-19, and (2c) immigration. Primary theme 3: lack of confidence, yet willingness to be vaccinated, with subthemes (3a) fear and distrust and (3b) willingness to be vaccinated. In summary, participants felt hesitant, although not completely opposed, to receiving COVID-19 vaccinations due to the information they gathered on vaccines from various sources received in the context of important personal factors (e.g., immigration, underlying health concerns, etc.).

Conclusions: Overcoming vaccine hesitancy in vulnerable populations such as the Hispanic communities may require addressing issues of message clarity through trusted sources while considering personal contextual factors. Healthcare professionals can begin by initiating discussions with patients to understand individual circumstances and concerns and provide information on COVID-19 vaccines that clarify areas of confusion.

## Introduction

As of November 2022, 84.3% of Hispanic adults reported completing the primary series of vaccination against COVID-19 [1]. While disparities in vaccination rates have continued to improve, studies have consistently identified vaccine hesitancy within racial/ethnic subgroups. However, few studies have aimed to understand why lower rates of COVID-19 vaccine uptake exist among Hispanic/Latinx individuals [2]. Additionally, language and cultural barriers make studying vaccine hesitancy in minority groups particularly challenging. Nevertheless, understanding beliefs about COVID-19 vaccines among Hispanic/Latinx individuals may help elucidate reasons for lower rates of COVID-19 vaccine uptake in this population. Understanding potential causes for vaccine hesitancy may become increasingly important as recommendations for COVID-19 vaccinations including booster series continue to evolve. Therefore, we aimed to investigate factors that influence COVID-19 vaccine attitudes among unvaccinated Hispanic/Latinx adults utilizing a free community clinic.

## Materials and methods

We conducted a qualitative study using semi-structured individual interviews with self-identified unvaccinated Hispanic/Latinx individuals to understand beliefs about COVID-19 vaccines. We used convenience sampling to recruit participants from a free community clinic in downtown Orlando, Florida, USA. Inclusion criteria were: 1) being >18 years old, 2) self-identifying as Hispanic/Latinx, 3) being a resident of Orlando, and 4) having not received a COVID-19 vaccine. Participants were excluded if they did not speak Spanish or English, had medical or psychiatric conditions that precluded them from answering interview questions or had previously received a COVID-19 vaccine.

Interviews were conducted from May to July 2021, individual interviews lasted 15 to 60 minutes and were conducted in Spanish or English. Interviews were conducted using an interview guide that was developed by authors TK, LT, and CH, using the constructs of the Health Belief Model which include perceived susceptibility, perceived severity, perceived benefits, perceived risks, and cues to action [3]. Additionally, participants were also asked about their general understanding and perception of vaccines, including non-COVID vaccines. For each question, participants were asked open-ended questions and subsequent follow-up questions to allow participants to elaborate on their initial responses. Interview questions, including those pertaining to the Health Belief Model, are presented in Table 1.

**Table 1:** Semi-structured interview guide This is the interview guide used to conduct semi-structured interviews. Open-ended questions (left-hand column) were used to initiate discussions while probing/closed-ended questions (right-hand column) were used to clarify or expand upon points made by participants based on the participant's response to the open-ended question.

Open-ended Questions	Follow-up/Probing Questions
What does the word vaccine mean to you?	Can you please elaborate on what you mean by (…)?
In general, what are your thoughts or feelings toward vaccines?	Can you please elaborate on what you mean by (…)?
Would you describe your feelings towards vaccines? (e.g., positive, neutral, or negative)?	Can you please elaborate on why you feel this way (positive, negative, neutral, or otherwise)?
In general, what are your thoughts/feelings about COVID-19 vaccines?	How would you describe your feelings toward the COVID-19 vaccines (e.g., positive, neutral, or negative)? Can you please describe how you came to feel this way about the COVID vaccine?
Do you believe you feel differently towards the COVID-19 vaccine compared to other vaccines that are available like influenza, hepatitis, or HPV?	Why do you believe your feelings about COVID-19 vaccines differ from others?
Questions Based on the Constructs of the Health Belief Model	
Perceived Susceptibility: Do you believe you are susceptible to contracting COVID-19? If you feel comfortable, sharing, have you or a family member/close friend been infected with COVID before?	Please elaborate on why you believe that you are (or are not) susceptible to COVID-19
Perceived Severity: Are you worried about the severity of symptoms/complications of contracting COVID-19?	Please elaborate on why you are (or are not) concerned about the potential severity of getting COVID-19
Perceived Benefits: What do you believe are the benefits of the COVID-19 vaccine? What do you believe are the risks of the COVID-19 vaccine?	What do you believe are some good things that can come from getting the COVID-19 vaccine? Do you think that there are any risks when getting the COVID vaccine?
Perceived Barriers: Do you feel that there are any barriers that would keep you from getting a COVID-19 vaccine?	Would it be challenging to get the vaccine? Why? Can you describe what you believe those barriers are? What barriers prevent you from getting a vaccine?
Cues to Action (information, people, events influencing action): Where do you prefer to get information about the COVID-19 vaccine (e.g., family, internet, etc.)?	Whose opinion do you trust for getting information about COVID-19 or its associated vaccine? Why did you choose this source for information? Can you describe how this source has impacted your attitudes toward the COVID vaccine?

After obtaining consent, interviews were audio-recorded using an audio-recording device. Audio recordings were transcribed verbatim using Sonix automated transcription software (Sonix AI, San Francisco, CA, USA) [4]. Transcript outputs for each interview were reviewed by two bilingual members of the study team to assure consistency with audio recordings and any discrepancies were discussed until a consensus was reached. Interviews that had been transcribed to Spanish were then translated to English using Systrans Pro (San Diego, CA, USA) [5]. Translated interviews were then independently reviewed by two bilingual members of the study team and an additional certified academic Spanish translator who was not associated with this study. Both Sonix and Systrans software are accepted as industry standard tools that produce quality outputs that can be used for industry and scientific research. 

Transcribed interviews were then reviewed and recurrent themes in participant responses were coded by TK using inductive content analysis methodology. Inductive content analysis entails identifying codes and themes without using a predetermined framework. This approach is typically used when there are a limited number of studies and pre-existing theories that explore a phenomenon [6]. Given the limited qualitative research on COVID-19 vaccine hesitancy among racial and ethnic minorities, inductive content analysis was chosen as the preferred qualitative analysis method to identify recurring themes. Interview coding was completed using Dedoose Qualitative Analysis software (Manhattan Beach, CA, USA) to identify recurring themes [7]. Coded portions of interviews representing recurring themes were then grouped into primary themes and subthemes by two independent reflecting the identified themes. Any discrepancies were discussed, and a consensus was established. Interviews were conducted and analyzed until thematic saturation was reached, which was determined to have been achieved when further observation and analysis revealed no new themes [8]. Primary themes, subthemes, and representative quotes are presented in the results section. This study was approved by the University of Central Florida Institutional Review Board (approval no. STUDY00002703) and the study protocol was reviewed and approved by the Shepherd's Hope Downtown Orlando clinic administration prior to study initiation. 

## Results

**Figure 1:**
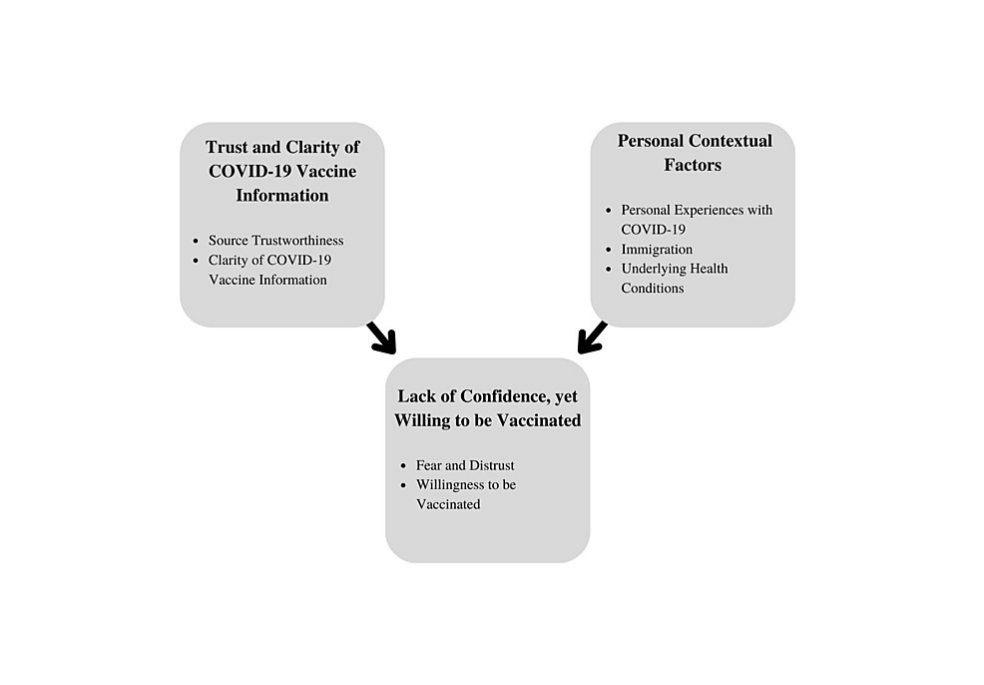
Schematic depicting the relationship between the identified primary themes (bolded headers of individual boxes) and respective subthemes (bulleted) within each primary theme

Our sample included 20 adults aged 21 to 73. Most participants were female (n=13), and from Venezuela (n=11), Colombia (n=3), and Puerto Rico (n=3). Qualitative analysis of participant responses revealed three primary themes and respective subthemes within each primary theme (Figure 1). Primary theme 1: trust and clarity of COVID-19 vaccine information with subthemes (1a) source trustworthiness, and (1b) clarity of COVID-19 vaccine information. Primary theme 2: personal contextual factors with subthemes (2a) underlying health conditions, (2b) personal experiences with COVID-19, and (2c) immigration. Primary theme 3: lack of confidence yet willingness to be vaccinated with subthemes (3a) fear and distrust, and (3b) willingness to be vaccinated. We present the primary themes, subthemes, and illustrative quotes gathered from qualitative analysis in Table 2.

**Table 2:** Primary themes, subthemes, and illustrative quotes from participant responses

Primary Theme	Subthemes	Illustrative Quotes on Participant Nationality, Sex, and Age
1. Trust and Clarity of COVID-19 Vaccine Information	(1a) Source Trustworthiness	“I get information (about COVID-19 vaccines) from a friend who works with the mayor. So, I feel like I am well informed.” Participant 1: Venezuelan female, age 35 | “The most important thing is recent statistical data (about COVID-19 vaccines) from scientists.” Participant 7: Venezuelan Female, age 41 | “I read a lot of news, but I would say that I trust medical blogs (for information about COVID-19 vaccines) more than traditional media.” Participant 16: Venezuelan Female, age 26
	(1b) Clarity of Information	“I’ve heard that the side effects (from COVID-19 vaccines) are uncommon, but I don’t really know.” Participant 4: Venezuelan Male, age 40 | “Scientific studies say that few people (who get COVID-19 vaccines) die. But I don’t know anyone who has died so I don’t know what that is about.” Participant 8: Venezuelan Female, age 73 | “What makes me confused is that I've read that the virus is mutating.” Participant 7: Venezuelan Female, age 41
2. Personal Contextual Factors	(2a) Underlying Health Conditions	“If I were a person that didn't have this disease (lupus), I would definitely get vaccinated right away.” Participant 11: Colombian Female, age 28 | “I was told I got shingles because my immune system is weakened. So, I'd rather wait a little longer (to get a COVID-19 vaccine).” Participant 18: Venezuelan Female, age 44 | “If I didn’t have that kind of problem (cardiovascular disease) I'd already have gotten it (COVID-19 vaccine). That’s my fear.” Participant 12: Venezuelan Male, age 66
	(2b) Personal Experience with COVID-19	“I have seen a lot of people who have been infected with that virus (COVID-19) that have died.” Participant 10: Venezuelan Female, age 65. | “I've heard there can be very bad repercussions from contracting it (COVID-19) but personally, I don't worry about it.” Participant 13: Puerto Rican Male, age 54 | “One benefit is protection (from COVID-19 vaccines) so that you will not get infected with COVID.” Participant 3: Venezuelan Female, age 39
	(2c) Recent Immigration	“In this country (United States), I don’t see any barriers to getting the vaccine. But in my country (Venezuela), it hasn’t even arrived yet.” Participant 6: Venezuelan Male, age 47 | “They make it difficult (to get a COVID-19 vaccine) because they require you to give them some documentation.” Participant 14: Guatemalan Male, age 41 | “It would help if they told us what documents we needed (to get a COVID-19 vaccine).” Participant 5: Mexican Male, age 25
3. Lack of Confidence yet Willing to be Vaccinated	(3a) Fear and Distrust	“I'm scared because I don't know how I'm going to react (to the vaccine).” Participant 15: Peruvian Female, age 65 | “In my opinion, part of the pharmaceutical industry created this disease (COVID-19) and now it’s creating the cure (COVID-19 vaccines).” Participant 17: Colombian Female, age 52 | “I’m panicking about getting the (COVID-19) vaccine because I have relatives who got the second dose and have died.” Participant 9: Colombian Female, age 50
	(3b) Willingness to be Vaccinated	“The only thing that would convince me to get it (a COVID-19 vaccine), would be if they told me they were 100% certain I wouldn’t get infected.” Participant 2: Venezuelan Female, age 52 | “I’m not going to get it (a COVID-19 vaccine) unless they require it for travel or work.” Participant 19: Puerto Rican Female, age 21 | “I may get it in the future because everyone (will have) finished getting it. There is no doubt about that. But not right now.” Participant 20: Puerto Rican Male, age 22

Trust and clarity of COVID-19 vaccine information

Source Trustworthiness

Most participants cited using more than one preferred information source when seeking information about COVID-19 vaccines. However, not all information sources were viewed as equally trustworthy. Government (local or federal)-based entities and scientific professionals (physicians or scientists) were viewed as trustworthy sources of information, while traditional news outlets and social media were perceived as less reliable.

Clarity of COVID-19 Vaccine Information

Commonly encountered information about COVID-19 vaccines included adverse reactions, including vaccine-related mortality, vaccine development time, and removal of vaccines from consumer markets. Even when information on these topics came from reliable sources, participants found the information they received unclear. Participants often had questions such as: “How many people will have a reaction to the vaccine?” and “What does it mean when they say the virus is mutating?”

Personal contextual factors

Underlying Health Conditions

Participants cited their underlying health conditions as a factor that influenced their attitudes toward COVID-19 vaccines. Conditions ranged from diagnosed syndromes such as lupus and hypertension to unspecified health issues such as having an “overwhelmed immune system” and “cardiovascular” issues. Participants expressed beliefs that these underlying health conditions might predispose them to experience significant adverse reactions to COVID-19 vaccines, such as significant flu-like symptoms, myocarditis, or even an increased risk of death following vaccination.

Personal Experiences with COVID-19

Personal experience with COVID-19 included having personally contracted COVID-19 and having family or friends who had contracted the virus. Participants also reported variable severity of COVID-19 infections, which ranged from being asymptomatic to death. However, participants generally expressed concern about health effects secondary to COVID-19 infections and viewed COVID-19 vaccines as a form of protection from COVID-related health sequelae.

Immigration

Immigrant status had a variable influence on participant beliefs toward COVID-19 vaccines. Participants expressed gratitude that high-quality COVID-19 vaccines were readily available in the U.S., in comparison to their home countries. However, for some participants, immigration status was cited as a primary barrier to vaccination due to lacking proper documentation to obtain a vaccine.

Lack of confidence, yet willing to be vaccinated

Fear and Distrust

Participants expressed fear and distrust of COVID-19 vaccines. Fear was often reported in connection to vaccine side effects, including vaccine-related mortality. Participants often referred to side effects as a vague entity, but in some cases, specific examples that were provided included fevers, headaches, blood clots, neurologic effects, and myocarditis. Information about adverse effects was gathered from a variety of sources including anecdotes from family and friends as well as news outlets and social media. In instances where mortality was attributed to an adverse effect of COVID vaccines, anecdotes were typically gathered from friends or family members, usually in the participant’s country of origin with few other details provided. Additionally, participants’ distrust of COVID-19 vaccines was generally associated with concerns about vaccine development time, citing that to participants the time from development to distribution seemed short according to their understanding. Furthermore, this distrust was generally directed toward vaccine stakeholders (e.g., vaccine-producing companies) and news outlets citing that these entities would benefit from the increasing distribution of vaccines, causing participants to feel skeptical about possible ulterior motives.

Willingness to Be Vaccinated

Although participants felt wary about getting COVID-19 vaccinations, for most, this did not preclude them from possibly getting vaccinated in the future. Willingness to be vaccinated was contingent on certain changes, including the resolution of health issues, requirements by employers, and receiving more data about vaccines.

## Discussion

The overall attitudes toward COVID-19 vaccines in this study sample may best be described as “cautious acceptors,” as described by Olson et al., which refers to individuals who may hold fears about being vaccinated but are still willing to accept vaccines [9]. This classification is useful in that it may help identify an individual’s or group’s propensity for vaccine uptake. However, such a descriptor does not elucidate the underlying reasons that these individuals are “cautious”, and therefore may have limited utility in trying to identify the basis of an individual’s or group’s vaccine hesitancy.

The findings presented in this report support those drawn from similar qualitative studies. In a qualitative study of African American and Hispanic adults in Connecticut, Balasuriya et al. concluded that information about COVID-19 vaccines from “consistent messaging via trusted messengers,” may act as a positive driver of COVID-19 vaccine acceptance [10]. Such “trusted messengers” appear to be individuals or entities that are intimately connected to the communities that participants belong to. The findings present in this study appear to support the idea from previous studies that local healthcare professionals and public health entities may play an important role as trusted messengers of reliable information on COVID-19 vaccines in this sample of Hispanic adults. However, based on the results presented here, the role of family members and friends as messengers of vaccine information should not be dismissed by public health efforts aiming to address COVID-19 vaccine hesitancy.

In this study sample, it appears that COVID-19 vaccine hesitancy may have been eased by providing information that addressed specific concerns within this group, including vaccine tolerability in patients with underlying health conditions and immigration documentation requirements for vaccination sites. These findings, as well as those from prior studies, suggest that healthcare professionals and public health entities influence beliefs toward COVID-19 vaccines by addressing population-specific and engaging in meaningful conversations where patients can express personal concerns freely. However, given the small sample size and limited available literature, the degree of local or regional variability in these concerns remains unknown and merits further investigation.

This study has certain limitations. The most important of which is the small sample size. Thus, the results of this study cannot be appropriately generalized to larger Hispanic populations. Convenience sampling creates a risk of sampling bias and interview responses may be subject to social desirability responses. Additionally, as these data were collected earlier in the COVID-19 pandemic, this study does not account for how these beliefs may have changed over time. To address these limitations, future survey-based studies may assess the generalizability of these findings to other Hispanic/Latinx subgroups and elucidate how beliefs toward COVID-19 vaccines vary over time.

## Conclusions

In this study, trust and clarity of COVID-19 vaccine information and personal experiences such as an individual’s perceived health problems, influenced participant belief about COVID-19 vaccines. A lack of confidence, yet an overall willingness to accept these vaccines, characterized these beliefs. Further studies are needed to understand the generalizability of these findings among other Hispanic populations and how these beliefs may change over time with the continually evolving circumstances of the COVID-19 pandemic. 
